# Action of *Mangifera indica* Leaf Extract on Acne-Prone Skin through Sebum Harmonization and Targeting *C. acnes*

**DOI:** 10.3390/molecules27154769

**Published:** 2022-07-26

**Authors:** Morgane De Tollenaere, Cloé Boira, Emilie Chapuis, Laura Lapierre, Cyrille Jarrin, Patrick Robe, Catherine Zanchetta, David Vilanova, Bénédicte Sennelier-Portet, Jessy Martinez, Amandine Scandolera, Daniel Auriol, Romain Reynaud

**Affiliations:** 1 Givaudan Active Beauty, R&D, 51110 Pomacle, France; cloe.boira@givaudan.com (C.B.); emilie.chapuis@givaudan.com (E.C.); laura.lapierre@givaudan.com (L.L.); amandine.scandolera@givaudan.com (A.S.); 2 Givaudan Active Beauty, R&D, 31400 Toulouse, France; cyrille.jarrin@givaudan.com (C.J.); patrick.robe@givaudan.com (P.R.); catherine.zanchetta@givaudan.com (C.Z.); daniel.auriol@givaudan.com (D.A.); romain.reynaud@givaudan.com (R.R.); 3 Genomic Tales, Consulting, AD200 Encamp, Andorra; david.vilanova@givaudan.com; 4 Givaudan Active Beauty, R&D, 84911 Avignon, France; benedicte.sennelier-portet@givaudan.com (B.S.-P.); jessy.martinez@givaudan.com (J.M.)

**Keywords:** Acne, microbiota, *C. acnes*, lipase activity, seboregulation

## Abstract

(1) Background: Preclinical studies report that the ethanolic fraction from *Mangifera* *indica* leaves is a potential anti-acne agent. Nevertheless, the biological activity of *Mangifera indica* leaves has scarcely been investigated, and additional data are needed, especially in a clinical setting, for establishing the actual effectiveness of *Mangifera indica* extract as an active component of anti-acne therapy. (2) Methods: The evaluation of the biological activity of *Mangifera indica* extract was carried out through different experimental phases, which comprised in silico, in vitro, ex vivo and clinical evaluations. (3) Results: In silico and in vitro studies allowed us to identify the phytomarkers carrying the activity of seboregulation and acne management. Results showed that *Mangifera indica* extract reduced lipid production by 40% in sebocytes, and an improvement of the sebum quality was reported after the treatment in analyses performed on sebaceous glands from skin explants. The evaluation of the sebum quantity and quality using triglyceride/free fatty acid analysis conducted on Caucasian volunteers evidenced a strong improvement and a reduction of porphyrins expression. The *C. acnes* lipase activity from a severe acne phylotype was evaluated in the presence of *Mangifera indica*, and a reduction by 29% was reported. In addition, the analysis of the skin microbiota documented that *Mangifera indica* protected the microbiota equilibrium while the placebo induced dysbiosis. (4) Conclusions: Our results showed that *Mangifera indica* is microbiota friendly and efficient against lipase activity of *C. acnes* and supports a role for *Mangifera indica* in the therapeutic strategy for prevention and treatment of acne.

## 1. Introduction

Acne is the most common chronic inflammatory disease of the skin. Although it is not a life-threatening or physically debilitating disease, it can cause substantial discomfort and pain, impacting patients’ quality of life [1,2].

Increased sebum secretion rate is the major feature in the pathophysiology of acne. Along with the increased secretion, quantitative and qualitative modifications of sebum are likely to occur [3]. In addition, the hair follicle can be infected with bacteria, which triggers an immune reaction. Among them, *Cutibacterium acnes* (*C. acnes*, formerly known as *Propionibacterium acnes*) is considered as the main bacterium responsible for acne [4]. For a few years, the involvement of *C. acnes* in acne was controversial and thorough investigations demonstrated that higher representation of phylotype IA1 was detected on acne lesions rather than healthy skin. Genomic sequencing and characterization evidenced that the pathogenicity of *C. acnes* was more related to its lipolytic activity than its proliferation capacity [5,6].

Therefore, therapeutic approaches focus on the main factors implicated in acne, namely the androgen-mediated sebogenesis, hyperkeratinization, colonization with *C. acnes* and inflammation related to innate and adaptive mechanisms [7]. Current treatments depend on the severity of acne and usually vary from topical to oral systemic therapies. 

The most common topical approach is a combined treatment of a retinoid, mainly isotretinoin, as the only agent that can affect all four main factors related to acne, and benzoyl peroxide, which is highly effective in reducing both sensitive and resistant strains of *C. acnes* [8,9]. Unfortunately, these kinds of therapies can cause adverse reactions [10,11,12]. In addition, considering the importance of minimizing the risk of community antibiotic resistance, the development of non-antibiotic therapies is preferable [13].

*Mangifera indica* L. Anacardiaceae, commonly known as mango, belongs to the Anacardiaceae plant family and is widely grown in tropical regions, especially in India and in Thailand. *M. indica* has been an important medicinal plant in the Ayurvedic and indigenous medical systems for over 4000 years [14]. Its parts are commonly used in folk medicine as remedies for various disease conditions and especially the leaves, which are traditionally known to be useful for infections, burns, and scalds, are linked to antioxidant, antimicrobial, antiviral, and antibacterial activities since they have a high polyphenol content [15,16,17]. 

Indeed, polyphenols are the main identified compounds in mango and mangiferin, a C-glucoside xanthone, is the major phytomarker of all the parts of mango (peel, leaves, twigs, bark) [18,19]. Mangiferin is widely distributed in higher plants where it provides protection against different forms of static and dynamic stresses including pathogenic microorganisms [20]. Moreover, preclinical *in tubo* studies have shown that the ethanolic fraction obtained from *Mangifera indica* leaves and kernel is a potential anti-acne agent due to its inhibition activity against *C. acnes*, as well as its potent free-radical scavenging and inhibitory effects on acne-related pro-inflammatory cytokines [21,22,23].

Nevertheless, the biological activity of *Mangifera indica* leaves has scarcely been investigated, and additional data are needed, especially in a clinical setting, for establishing the actual effectiveness of *Mangifera indica* extracts as active components of anti-acne therapy [15,16,21].

The present study aims to provide preclinical and clinical evaluations about the biological activity of *Mangifera indica* leaf extract against *C. acnes* and to evaluate the implications of this activity in the treatment and prevention of acne.

## 2. Results

*Mangifera indica* extract was characterized by HPLC-UV and HPLC-MS (data not shown) and four main compounds were identified: mangiferin, penta-O-galloyl-beta-D-glucose, iriflophenone-3-C-beta-glucoside and maclurin-3-C-beta-glucoside. The results confirmed previous research already published on the composition of *M. indica* leaves [24,25].

*Mangifera indica* extract and its main phytomarker, mangiferin, were evaluated for seboregulation. The seboregulatory activity was evaluated using an in vitro lipogenesis inhibition model on a sebocyte cell line. Stimulated conditions by a lipogenic mix were used to mimic the condition of sebum excess.

### 2.1. In Vitro Seboregulation Activity of Mangifera indica Extract at 0.3%

**Figure 1:**
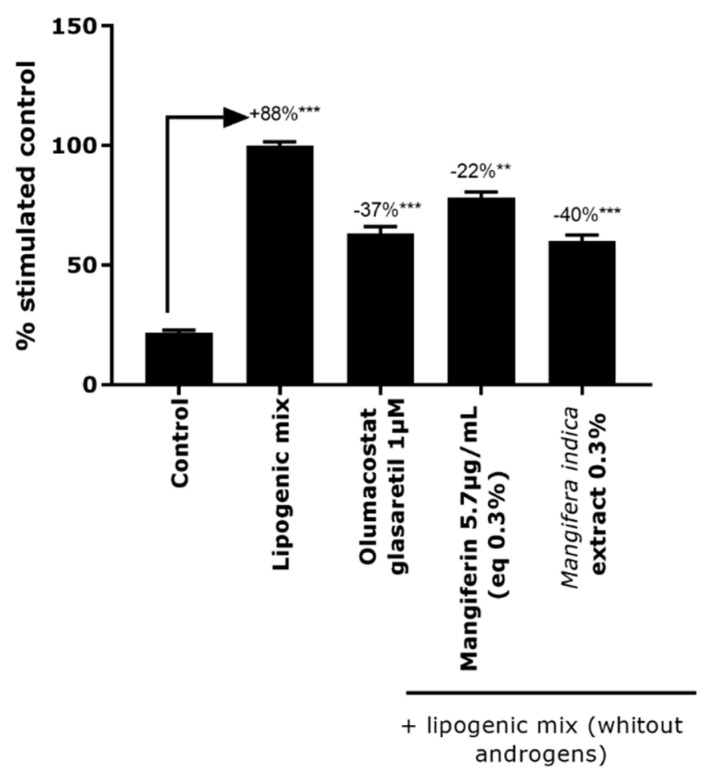
Lipid accumulation analysis measured by quantification of fluorescence intensity Bodipy^®^. The results are expressed in percent of lipogenic mix condition. One-way ANOVA multiple comparisons test was used to determine the significance of the data with ** *p* < 0.01 and *** *p* < 0.001.

A significant increase in lipid content was observed after 7 days of lipogenic mix exposure. After 7 days of treatment with *Mangifera indica* extract at 0.3%, the lipid content percentage was significantly reduced by 40% (Figure 1). Mangiferin, the main phytomarker identified in the *Mangifera indica* extract, demonstrated a significant reduction of lipid content by 22%. The reduction of lipid synthesis was not equivalent to the one obtained in presence of *Mangifera indica* extract, leading us to the conclusion that the mangiferin does not carry by itself the entire efficacy of the extract. Other phytochemical compounds seemed to be involved in the seboregulation. A similar activity was observed in the positive reference, as shown by the reduction of lipid production by 37%.

### 2.2. In Silico Studies: Comprehension of Mode of Action of the Three Main Phytochemical Compounds Found in Mangifera indica Extract

Inverse docking was performed for maclurin and iriflophenone, two phytochemical compounds found in *Mangifera indica* extract (Figures S1 and S2). The objective of this study was to have more understanding about their mechanisms of action and their involvement in seboregulation.

The inverse docking allows the identification of potential receptor/proteins interactions using a prediction based on structural homology with known molecules in a data basis. A score over seven is considered as significant and robust enough to consider this interaction as possible.

Various proteins involved in the modulation of lipogenesis such as PPAR family or Retinoic family were identified as targets for both iriflophenone and maclurin (list of targets and scores are detailed in Supplementary Materials). Moreover, the analysis demonstrated that iriflophenone and maclurin can interact directly with enzymes involved in sebum production such as FDA synthase, squalene synthase and others.

**Figure 2:**
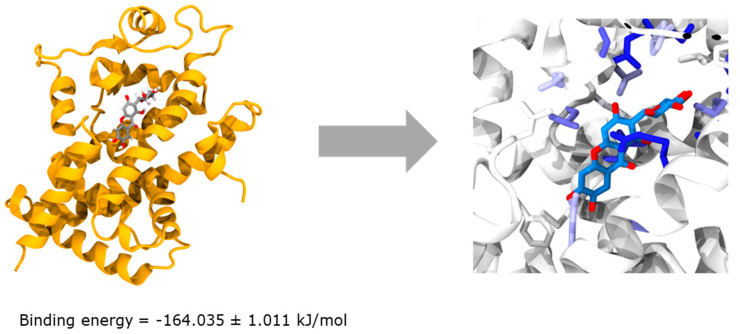
Representation of the binding energy relative to the interaction between mangiferin and PPARγ (**left**) with the identification of engaged residues in the interaction (**right**).

**Figure 3:**
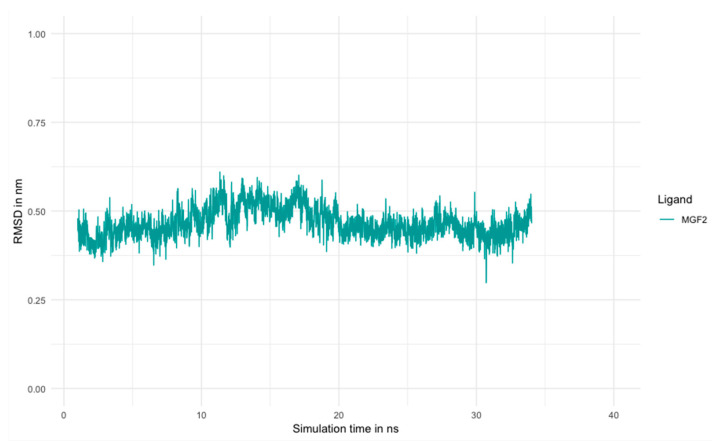
Illustration of the result of molecular dynamics demonstrating the evolution of the distance between residues during the time of stimulation (35 ns).

To further understand the mode of action, the interaction between the main phytomarker mangiferin and the transcription factor PPARγ, which has been well described for its involvement in lipogenesis modulation, was evaluated. The molecular docking revealed a very low binding energy at −164 kJ/mol, indicating a possible interaction between the two molecules as shown in Figure 2. No unfavorable residues were found, demonstrating an interaction without any constraint. Additionally, the stability of the interaction using molecular dynamic simulations was measured for 35 ns. The distance between residues of both structures was stable during the entire stimulation as shown in Figure 3.

### 2.3. Ex Vivo Seboregulation Activity of Mangifera indica at 1%

**Figure 4:**
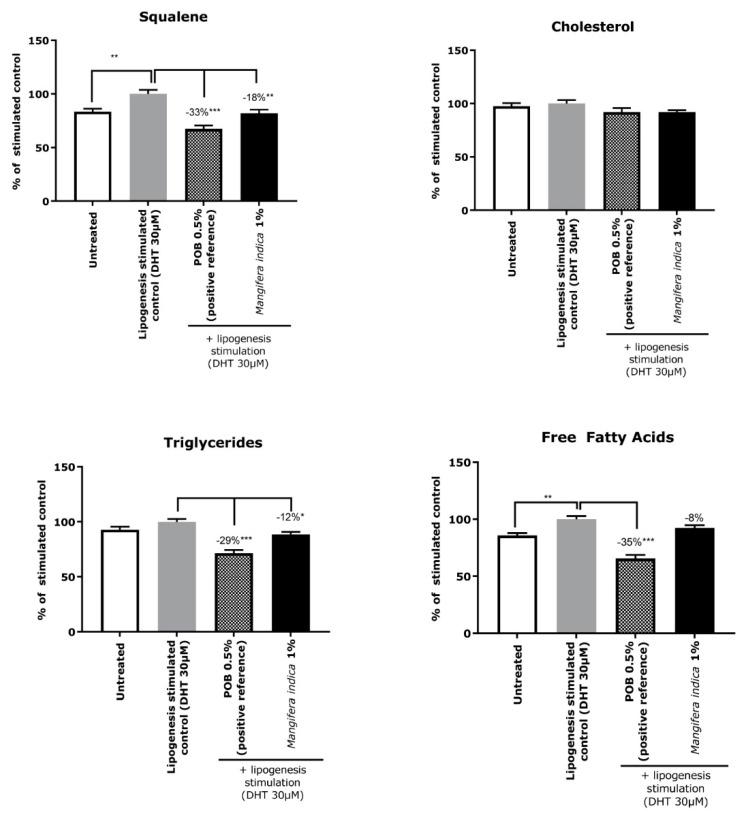
Lipids extracted from sebaceous glands and quantified by GC/MS or LC/MS. The results are expressed in percent of the stimulated control (Untreated + DHT 30 µM). ANOVA followed by Dunnett’s multiple comparisons test was used to determine the significance of the data with * *p* < 0.05, ** *p* < 0.01 and *** *p* < 0.001.

The lipogenesis stimulation induced a significant increase in squalene and FFA content. There was no effect on the cholesterol family of compounds (Figure 4). 

Results with *Mangifera indica* extract showed a significant percentage reduction of triglyceride (−12%) and squalene contents (−18%). The reduction in FFA was 8% but not significant (Figure 4).

The positive reference showed a significant reduction in each lipid family except for the cholesterol one (Figure 4). 

### 2.4. Impact of Mangifera indica 1% on Sebum

**Figure 5:**
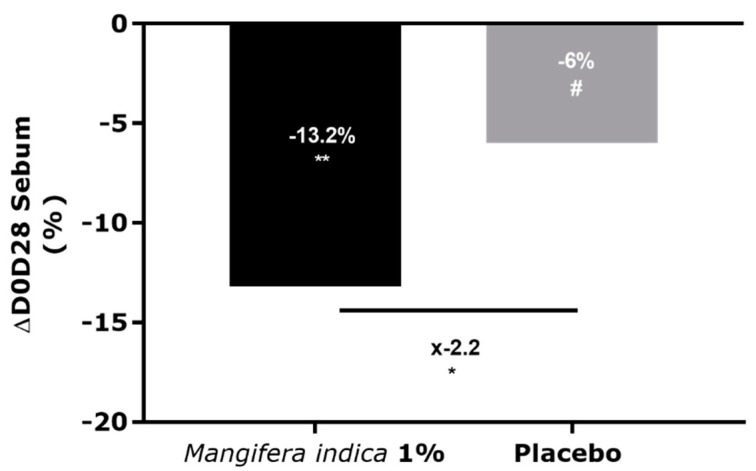
Sebum analysis by Sebumeter^®^ on Caucasian volunteers after 28 days of application of cream containing *M. indica* extract at 1% or placebo. Significance with # *p* < 0.1, * *p* < 0.05 and ** *p* < 0.01.

Results showed a decrease of sebum of −13.2% after 28 days of application with a cream containing *Mangifera indica* at 1%. A significant difference in the sebum content was observed between the placebo and extract-treated groups and was −2.2-fold in favor of the active group (Figure 5).

### 2.5. Impact of Mangifera indica on Skin Microbiota and C. acnes

#### 2.5.1. Analysis of TG/FFA Ratio in Volunteers Sebum

The analysis of TG/FFA can be used as a first indicator of microbiota metabolism and especially *C. acnes* since it is able to metabolize triglycerides into free fatty acids as a source of energy. Consequently, an impact on TG/FFA ratio may indicate a potential effect on *C. acnes*. 

**Figure 6:**
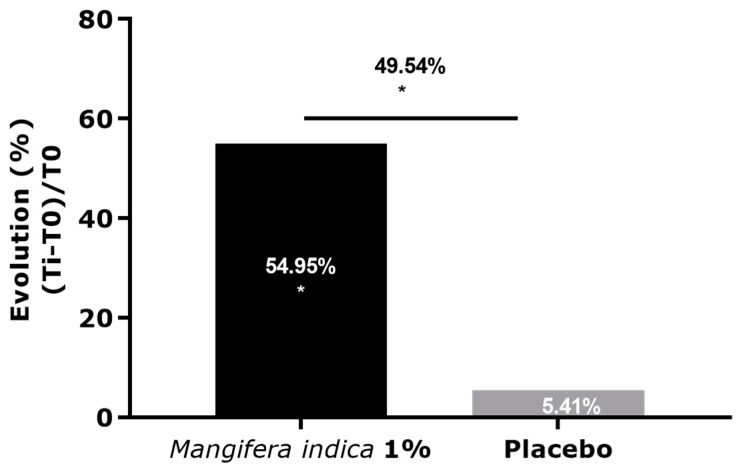
Analysis of TG/FFA ratio by GC LC/MS extracted from sebum of Caucasian volunteers after 28 days of application of cream containing *M. indica* extract at 1% or placebo. Significance with * *p* < 0.05.

Results showed an increase of TG/FFA ratio overtime of +55% after 28 days of application with a cream containing *Mangifera indica* at 1%. A significant difference in the TG/FFA ratio was observed between the two treatments and was equal to +49% in favor of the extract (Figure 6).

The increase of triglycerides and decrease of free fatty acids suggested a potential effect of *Mangifera indica* extract on *C. acnes* metabolism leading to a lower metabolization of triglycerides.

To go further on this hypothesis, an analysis of lipase activity in presence of the extract was required.

#### 2.5.2. Evaluation of *C. acnes* Metabolism Activity in Presence of *Mangifera indica*

**Figure 7:**
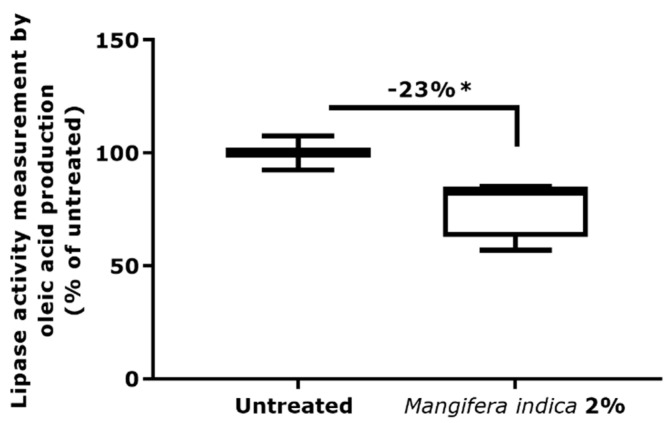
Measurement of lipase activity of representative severe acneic strain of *C. acnes* phylotype IA1 using oleic acid quantification in presence of *M. indica* extract at 2%. Significance with * *p* < 0.05.

To evaluate the hypothesis of the activity of *Mangifera indica* on *C. acnes* metabolism, the conversion of oleic acid from triolein was evaluated as an indicator of lipase activity. Results showed that the presence of *Mangifera indica* reduced the lipase activity of a representative severe acneic strain of *C. acnes* phylotype IA1 by 23% (Figure 7).

#### 2.5.3. Porphyrins Production 

**Figure 8:**
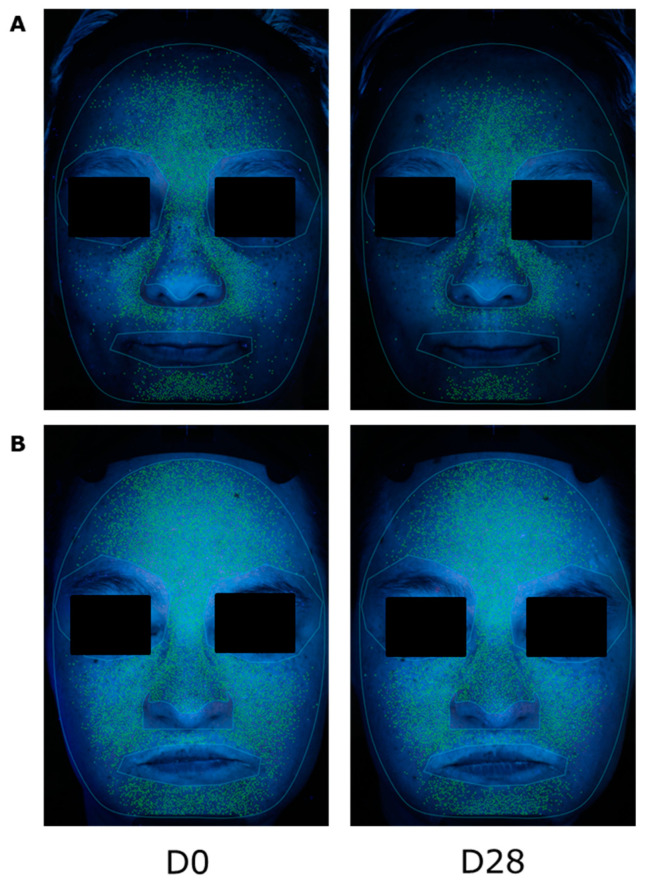
(**A**) Porphyrin detection on volunteer GP027 after application of *M. indica* at 1%. (**B**) Porphyrin detection on volunteer GP010 after placebo application.

As an indicator of the potential action of *Mangifera indica* on acne, porphyrins were quantified. *Mangifera indica* reduced porphyrins on the oily skin of the cheek and nose areas (Figure 8). In these areas, results showed a reduction of porphyrins intensity by 7% after 28 days of application with *Mangifera indica* 1%. The placebo showed a slight effect at the end of the treatment with a reduction by 3%. A significant difference of −2.4-fold between the two treatments was reported (Figure S3, Supplementary Materials).

#### 2.5.4. Impact of *Mangifera indica* 1% on Skin Microbiota Composition

**Figure 9:**
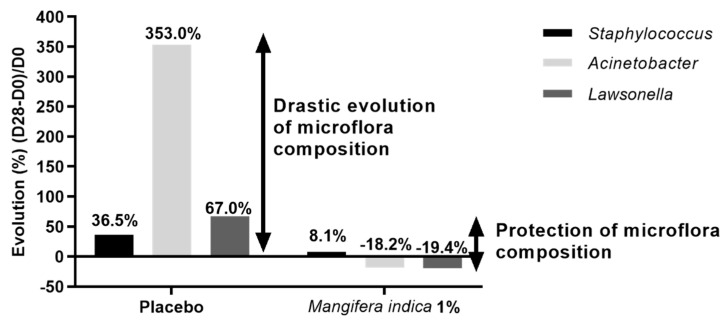
Representation of skin microbiota composition (analyzed by 16S rRNA sequencing) over time after application of placebo or *M. indica* extract at 1% after 28 days.

To evaluate if *Mangifera indica* had an impact on the skin microbiota composition, a metagenomic analysis was conducted. Results showed a significant increase of *Acinetobacter* (+353%) and S*taphylococcus* (+36%) genera proportion after 28 days of placebo application. During the same period, the relative abundances of these two genera did not statistically evolve with *Mangifera indica* extract 1% treatment (Figure 8). In addition, the impact of placebo on *Lawsonella* genus proportion after 28 days of treatment was not observed with *Mangifera indica* extract 1% (Figure 9).

## 3. Discussion

This work is among the first concerning the clinical evaluation of the biological activity of *Mangifera indica* leaf extract against *C. acnes*. In this study, *Mangifera indica* was able to significantly reduce lipid production up to 40%, as assessed using an in vitro human sebocytes model. Mangiferin, the main phytomarker of *Mangifera indica* extract, was tested within the same conditions of culture and the results evidenced a partial implication on its biological activity. To further understand the mode of action of the extract, inverse docking analyses were performed with iriflophenone and maclurin, the secondary phytomarkers identified. The analyses revealed that both compounds had an impact on PPAR and RXR families. Moreover, a potential interaction with enzymes involved in lipogenesis was suggested since FAD synthethase, squalene synthase and cholesterol oxidase obtained a score higher than seven. These results gave a more comprehensive explanation of how the extract modulates the lipogenesis and which phytomarkers are involved. Additionally, molecular docking between PPARγ and mangiferin confirmed the interaction and the mechanism of action of the phenolic compounds found in *Mangifera indica* extract.

Continuing on the evaluation of how *Mangifera indica* extract impacts sebum production, ex vivo analyses were performed using lift skin explants (a zone enriched in sebaceous glands), which were micro-dissected after treatment with *Mangifera indica*. Results reported an improvement of sebum quality after the treatment, as observed by the reduction of triglyceride and squalene contents, which were increased by lipogenic stimulation. Interestingly, these two families of lipids are significantly increased in the sebum from people with acne [3,26]. Thereby, it can be hypothesized that by reducing the quantity of sebum and improving its quality, the skin condition can be restored to normal.

The evaluation of the sebum quantity and quality using TG/FFA analysis conducted on Caucasian volunteers’ skin showed a strong improvement with *Mangifera indica*, as observed by a decrease of sebum content on the nose and cheek areas with −2.2-fold in favor of the extract-treated group. Moreover, the lipids analysis revealed an increase of the ratio TG/FFA by 49% in comparison with placebo after 28 days. The increased TG/FFA ratio obtained with *Mangifera indica* suggested a potential impact on *C. acnes* metabolism since the conversion of TG to FFA is managed by *C. acnes* lipases. To test this hypothesis, the lipase activity from a strain representative of the severe acne *C. acnes* phylotype IA1 was evaluated in the presence of *Mangifera indica* extract and a reduction by 23% was reported. The lipase liberates free fatty acids from triglycerides which are more viscous than triglycerides. Subsequently, free fatty acids release contributes to the obstruction and inflammation of the pilosebaceous unit [27,28]. Therefore, the level of lipase activity is correlated with the virulence of *C. acnes*. Indeed, the phylotype related to severe forms of acne, IA1, showed a higher oleate-degrading lipase activity than phylotypes related to healthy skin [5,6,29]. The amount of porphyrin was also assessed, and a significant reduction of porphyrin level by −2.4-times in comparison with placebo after 28 days was reported. This can be directly related to the skin microbiota, especially *C. acnes*, which is known to express high levels of porphyrins [30,31]. Of note, a decrease in porphyrin levels in acne patients has been related to clinical improvement [32].

The analysis of the skin microbiota evidenced that the placebo (a basic cosmetic formulation) led to significant modifications of the proportions of three bacterial genera on the skin (*Staphylococcus*, one of the major genera found on the skin, and *Acinetobacter* and *Lawsonella*), while the presence of *Mangifera indica* maintained the microbiota balance. 

Consequently, our results support the hypothesis that *Mangifera indica* can act on *C. acnes* metabolism, more precisely on lipase activity, without influencing its abundance. Authors should discuss the results and how they can be interpreted from the perspective of previous studies and of the working hypotheses. The findings and their implications should be discussed in the broadest context possible. Future research directions may also be highlighted.

## 4. Materials and Methods

### 4.1. Extract Description

Harvesting of the mango leaves (*Mangifera indica* L., Anacardiaceae) was carried out in Africa, mainly in Burkina Faso after the fruiting period and the wet season (or in between). A total of 10% max of leaves were harvested to preserve the plant life cycle. At the lab scale, the leaves were dried after sun exposure and ground to a particle size of 1.5 mm. The crushed and dried leaves were then macerated with a mixture of propanediol and water (3:1) to a plant/solvent ratio of 8% for 4 h at 60 °C. Then, the plant parts were removed by centrifugation. The remaining solution was filtered on filter Filtrox reference AF31H (12-5 µm) followed by a sterilized filtration on filter Filtrox reference AF ST 145 (−0.3–0.1 µm). 

The dry extract (average of 2% (*w*/*w*)) was placed in a mixture of 1,3-propanediol and water as the carrier solvent final extract, for a final composition referenced here as “*Mangifera indica* extract”.

High Performance Liquid Chromatography with UV detection at 315 nm and 295 nm was used to characterize the extract. Details of the method used is given in Supplementary Materials.

Thus, the extract contains active phytochemical compounds, mainly polyphenols and particularly mangiferin, C-benzophenones and penta-*O*-galloyl-β-D-glucose (details of composition and quantification are reported in Supplementary Materials). 

Within this study, the evaluation of the biological activity of *Mangifera indica* extract was carried out through different experimental phases, which comprised in silico, in vitro, ex vivo and clinical evaluations. 

### 4.2. Understanding of Mode of Action of Phytochemical Compounds Found in Mangifera indica Extract

#### 4.2.1. In Vitro Lipogenesis Inhibition Assay

Human SEBO662AR sebocyte cell lines were seeded in 96-well plates (50,000 cells/well) and cultured for 24 h in a culture medium. The medium was then removed and replaced by assay medium containing or not (control condition) *Mangifera indica* at 0.3% and mangiferin alone at 5.7 µg/mL, corresponding to its equivalent dosage in the extract at 0.3% (representative value corresponding to an average of mangiferin quantification in various batches in *Mangifera indica* extract). Cells were pre-incubated for 4 h in these conditions. Treatment with Olumacostat glasaretil at 1 μM was used as a positive control. Then, the lipogenic mix (containing vitamin C, vitamin D3, insulin and calcium, and no androgen, BioAlternatives, Gencay, France) was added, and the cells were incubated for 7 days. Half of the medium was removed at mid-term, i.e., after 3 days of incubation, and the treatments were renewed (including lipogenic mix stimulation). A non-stimulated control condition was performed in parallel. All experimental conditions were performed in *n* = 3, except for stimulated control conditions in *n* = 6. 

The lipid content was analyzed using Bodipy^®^ labeling (ThermoFisher, France). A detailed description of this analysis is reported in Supplementary Materials. Briefly, the lipid droplets were labeled with a specific Bodipy^®^ fluorescent lipid probe. The acquisition of the images was performed using INCell Analyzer™ 1000 (GE Healthcare, Chicago, IL, USA). The fluorescence intensity was analyzed exclusively in the lipid droplets and was normalized to the total number of cells (integration of numerical data with the Developer Toolbox version 1.5 (Hong Kong), GE Healthcare software).

A preliminary study of cytotoxicity was performed on these cell lines in order to determine the doses used (data not shown).

#### 4.2.2. Inverse Docking on Maclurin and Iriflophenone

An inverse docking was performed in order to complete the investigations since mangiferin does not contribute to the entire seboregulation efficacy.

Ligand- and protein-based screening was achieved with an in-house tool called Selnergy, proprietary of Greenpharma. This consists of searching similar annotated ligands in a database of 1,000,000 known active products and docking the studied molecules on a database of 10,000 protein X-ray and homology modelling structures. The proteins are annotated for their therapeutic classes, protein classes, organism sources and type of models. The detailed methods are described in Do QT et al., 2005 [33].

#### 4.2.3. Molecular Docking Analysis on Mangiferin and PPAR-γ

The protein data bank database lists numerous crystallographic structures of the ligand binding domain (residues 207 to 476) of PPAR-γ alone, bound to small molecules, in the presence or absence of another partner, RXR-α. Among the interesting structures, we retained the PDB codes 1PRG, 2Q5S and 4F9M which have 2.2, 2.05, 1.9 Å resolution, respectively. These targets were prepared with the Schrodinger suite in order to correct any defects in the structure (protonation states, steric clashes, water molecules/cofactors removal) that could impact the sampling during the molecular docking step.

The ligand structure mangiferin corresponds to the one identified in Pubchem. It has also been prepared with the Schrodinger Suite. Conformational searches confirmed that the ligand is flat and does not exhibit a large conformational diversity, leading to a “quite rigid” structure. All the docking experiments were focused on the cavity that binds total and partial PPAR-γ agonists. The results obtained show that the quality of the interaction is dependent on the initial PPAR-γ structure and the presence or absence of a cofactor in the cavity. 

The interaction energy was then quantified with the MM-PBSA method at 3 stages of the simulation (between the 25th and 30th ns, 95th and100th ns, and finally between 145th and 150th ns of simulation). This method also decomposes the energy contribution of the residues surrounding the binding cavity to the global interaction. The Mean of Energy contribution and standard error are plotted for each residue of PPAR-γ.

### 4.3. Ex Vivo Lipogenesis Characterization

To evaluate the impact of *Mangifera indica* extract on the quality of sebum, the total lipid content and four families of lipids (squalene, cholesterol, free fatty acids (FFA) and triglycerides (TG)) were assessed on sebum produced by human sebaceous glands obtained through an ex vivo skin explant. Lipogenesis was stimulated with dihydrotestosterone (DHT) and was used to mimic the condition of excess of sebum. 

Skin explants from the lifting of three donors (mean age ± standard deviation: 57 ± 2) were treated for 5 days with DHT (stimulated condition) or left untreated (control condition). In parallel, skin explants were treated in the presence of 0.5% of dibenzoyle peroxide (POB, positive control) or with 1% *Mangifera indica* extract. Sebaceous glands were micro-dissected from epidermis. Sebum lipids were then extracted from the sebaceous glands collected for the qualitative and quantitative analysis of lipids of interest onto a GC/MS system. A detailed description of lipids extraction and characterization methodologies is reported in Supplementary Materials.

### 4.4. Clinical Investigations

#### 4.4.1. Study Design

A double-blind, placebo-controlled clinical trial was conducted to evaluate the impact of *Mangifera indica* extract at 1% on oily skin (sebum > 140 μg/cm^2^ at the forehead) of healthy Caucasian women. Volunteers were 30 women (mean age ± standard deviation: 30.7 ± 5.8 years), equally divided to receive a cream containing either *Mangifera indica* extract at 1% (*n* = 15) or placebo (*n* = 15). 

Volunteers were randomly divided to apply a cream containing *Mangifera indica* extract at 1% or a placebo cream twice a day for 28 days.

All the subjects participating in the study gave their informed consent signed at the beginning of the study. The study was conducted according to the guidelines of the Declaration of Helsinki. This study performed on cosmetic products was within the definition of article L. 5131–1v of the French Public Health Code is in accordance with Decree n° 2017-884 of 9 May 2017, modifying some regulatory requirements concerning research involving human subjects.

#### 4.4.2. Study Measures

At D0 (baseline) and D28 (end of treatment), the sebum quantity and quality and the presence of porphyrins were assessed. A metagenomic analysis to study the skin microbiota of the volunteers was also carried out during the same clinical evaluation.

- Sebum evaluation: sebum quantity on cheek and nose areas were measured by a Sebumeter^®^, which is a device composed of a box and a cassette. This cassette consists of a frosted film (matte and translucent). The cassette (film) is brought into contact with the area of interest for 30 s. The cassette is then inserted into the machine, which measures the transparency of the film through transmitted light and determines the sebum rate.
  Measurements were taken on the forehead on day 0 to select and include the volunteers in the study, with a sebum level greater than 140 μg/cm^2^ considered as oily.
- Sebum evaluation: the identification and quantification of the different classes of lipids (squalene, cholesterol and FFA) was realized with a GC system (7890A from Agilent) coupled with an MS system (5975C Inert XL EI/CI MSD from Agilent, Santa Clara, USA). A calibration curve was performed on squalene and cholesterol; semi-quantitative analysis was performed for the other biomarkers. The evaluation of the sebum quality focused on the ratio TG/FFA, which can reflect some indirect information related to the evolution of skin microbiota metabolism, especially on *C. acnes*. Indeed, *C. acnes* consumes the TG and releases FFA, which will be degraded as an energy source for its growth. Therefore, an alteration of TG/FFA ratio could indicate a potential impact on *C. acnes*.
- Metagenomic analysis: microbiota samples were collected from the volunteers using a non-invasive swabbing method, using sterile swabs moistened with a sterile solution of 0.15 M NaCl. Swabs were transferred at −20 °C and kept frozen until DNA extraction. Sampling was done before treatment (D0) and after 28 (D28) days of treatment, using a standardized procedure. DNA extraction was performed using the DNeasy PowerLyzer^®^ PowerSoil^®^ DNA Isolation Kit with Qiacube device (Qiagen, Hilden, Germany), with the following modifications. The tip of each swab was detached with a sterile surgical blade and transferred into a 1.5 mL tube containing 750 μL of bead solution. The sampled biomass was suspended by stirring and pipetting and then transferred to a bead beating tube. The remaining steps were performed according to the manufacturer’s instructions. According to the manufacturer’s instructions, DNA concentration was determined using the Qubit™ dsDNA HS fluorometric quantitation kit (Invitrogen, ThermoFisher Scientific, Courtaboeuf, France). An extensive description of 16S rRNA gene sequencing and data analysis is reported in Supplementary Materials.
- Porphyrins detection using VISIA^®^ CR2.3 (Canfield, Parsippany, NJ, USA): porphyrins are organic compounds produced by *C. acnes* and linked to acne severity [21]. The impact of *Mangifera indica* 1% on porphyrin intensity was evaluated in full-face or localized skin areas (cheek and nose) after 28 days of application. The repositioning took place directly on the data-processing screen using an overlay visualization of the images at each time of acquisition. The Visia^®^ CR 2.3 allows taking pictures with different illuminations and very rapid capture of images. A series of photos taken under multispectral imaging and analysis allows capturing visual information affecting the appearance of the skin.

### 4.5. Evaluation of the Anti-Lipase Activity of Mangifera indica Extract on C. acnes 

To verify if *Mangifera indica* extract was able to modulate the *C. acnes* metabolism by reducing its lipase activity, triolein was used as a substrate, and the production of oleic acid was considered as the result of this enzymatic reaction, which was evaluated in the presence or not of *Mangifera indica* extract at 2%. Details about the culture of *C. acnes* (IA1, ARCC 6919), treatment and oleic acid quantification are reported in Supplementary Materials.

### 4.6. Statistical Analysis

For in vitro and ex vivo studies, all results are presented as mean ± standard error of the mean of three independent triplicates. A Shapiro–Wilk test was used to verify whether the raw data followed a Gaussian distribution. In the case of normally distributed data, the mean values were compared with either an unpaired t test (two or fewer groups) or one-way ANOVA followed by post-hoc test (more than two groups). In the case of non-normally distributed data, a Kruskal–Wallis test followed by a Mann–Whitney U test was used for unpaired data.

In all cases, results were considered significant with *p* < 0.1 with #, *p* < 0.05 with *, *p* < 0.01 with ** and *p* < 0.001 with ***.

For the clinical study, a non-parametric paired Wilcoxon test was used to analyze the effects of time of application, and a non-parametric unpaired Mann–Whitney test was used for the comparison of products.

## 5. Conclusions

Overall, obtained results showed that *Mangifera indica* is beneficial for acne-prone skins by harmonizing the sebum production and by acting on *C. acnes* metabolism. Moreover, the extract is microbiota friendly while being efficient against lipase activity of *C. acnes*. Considering that *C. acnes* lipase activity is linked with acne severity, our results support a role for *Mangifera indica* in the therapeutic strategy for the prevention and treatment of acne. Therefore, further clinical investigations for the promotion and application of *Mangifera indica* for acne management are suggested.

## 6. Patents

WO 2022/090322 Al, Morgane De Tollenaere, Cyril Jarrin, Carole Lambert, Romain Reynaud, Amandine Scandolera, Bénédicte Sennelier-Portet. 2021.

## Data Availability

Data available on request due to restrictions e.g., privacy/ethics reasons. The data presented in this study are available on request from the corresponding author. The data are not publicly available due to GDPR compliance.

## Supplementary Materials

The following supporting information can be downloaded at: https://www.mdpi.com/article/10.3390/molecules27154769/s1, Figure S1: HPLC-UV chromatograms of Mangifera indica extract at 315 and 295 nm; Figure S2: chemical structure of the 4 main phytomarkers; Figure S3: Porphyrin intensity analysis by VISIA® CR 2.3 on Caucasian volunteers (cheek and nose areas) after 28 days of application of cream containing M. indica at 1% or placebo; Table S1: Results for maclurin analysis; Table S2: Results for iriflophenone analysis. Refs. [34,35,36,37] are cited in the Supplementary Materials.

## Sample Availability

Samples of the compounds are available from the authors.
