# Supplementary material for: Action of Mangifera indica Leaf Extract on Acne-Prone Skin through Sebum Harmonization and Targeting C. acnes
Source: Molecules. 2022 Jul 26;27(15):4769. doi: 10.3390/molecules27154769 (PMC9331558; doi:10.3390/molecules27154769)
Supplement: Supplementary file 1 [file molecules-27-04769-s001.zip › Supplementary data_19072022.pdf]

Action of *Mangifera indica* leaf extract on acne-prone skin through sebum harmonization and targeting *C. acnes*

SUPPLEMENTARY DATA

Characterization of phytomarkers in Mangifera indica extract by HPLC-UV

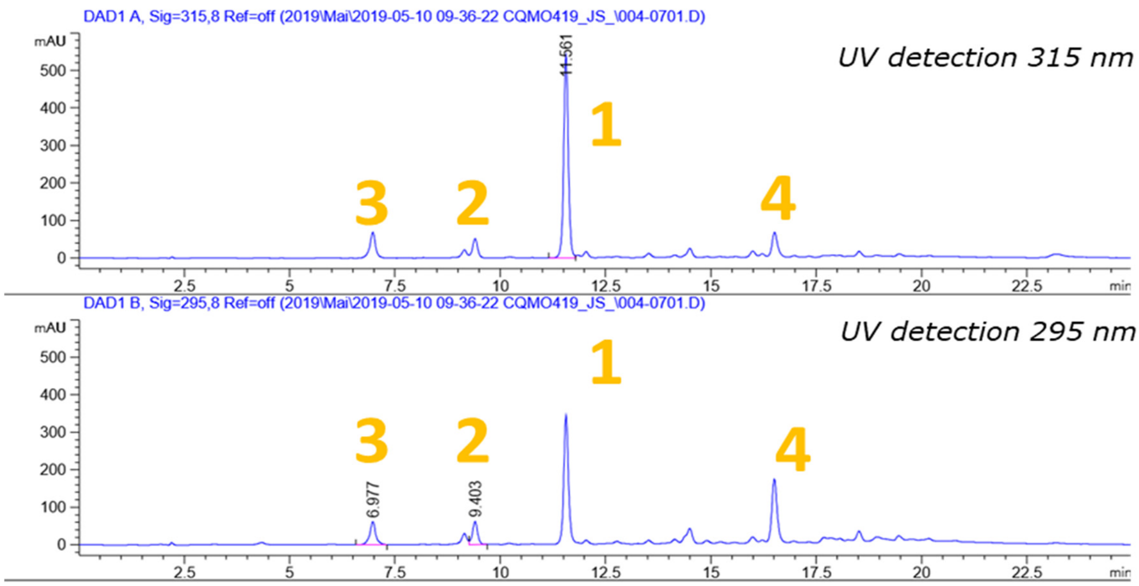

Figure S1: HPLC-UV chromatograms of Mangifera indica extract at 315 and 295 nm.

The compounds 1,2,3 and 4 were identified as follows:

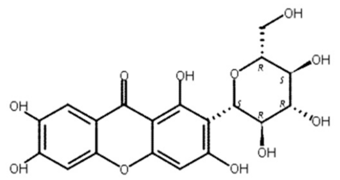

1-Mangiferin

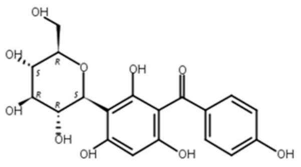

2-Irifluophenone-3C-β-glucoside

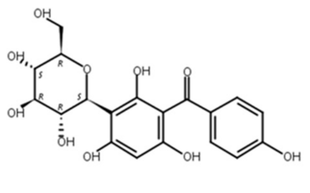

3-Maclurin-3C-β-glucoside

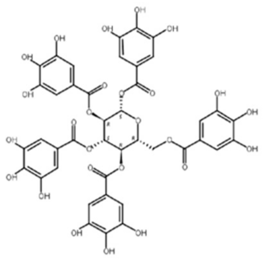

4-penta-O-galloyl-glucoside

Figure S2: chemical structure of the 4 main phytomarkers

The amount of the four main phytomarkers, which have been quantified in different batches as follows:

Batch	Mangiferin content (in ppm)	Iriflophenone-C-glucoside content (in ppm)	Maclurin-C-glucoside content (in ppm)	penta-O-galloyl-glucoside (in ppm)
a	1961.0	65.6.0	834.5	958.0
b	1896.0	434.3	278.2	816.0

Inverse docking

Table S1: Results for maclurin analysis

Category	Name	Score	Consequence
**Transcription factor / receptor**	RXR alpha	7.1674	Modulation of lipogenesis
	Nuclear receptor ROR-alpha	7.1393	
	Glucocorticoid receptor	7.4371	
	Insulin receptor	7.7369	
	IGF1	8.7891	
	PPARγ	7.7828	
	PPARδ	7.275	
**Enzyme**	Farnesyl pyrophosphate synthase	10.7054	Direct impact on specific proteins of sebum
	FAD synthetase	7.5232	
	Cholesterol oxidase	7.5104	
	Fatty acid oxidation complex subunit alpha	7.0973	
	Squalene synthase	7.0912	

Inverse docking

Table S2: Results for iriflophenone analysis

Category	Name	Score	Consequence
**Transcription factor / receptor**	PPARγ	9.4758	Modulation of lipogenesis
	PPARα	7.0902	
	PPARδ	7.0194	
	Retinoic acid receptor β	7.6238	

	Insulin receptor	7.3242	
	RXR α	7.1998	
**Enzyme**	FAD synthetase	8.4401	Direct impact on specific proteins of sebum (squalene ++)
	Squalene synthase	8.5685	
	Cholesterol oxidase	8.3317	
	Farnesyl pyrophosphate synthase	9.0946	
	Farnesyltransferase/geranylgeranyltransferase type-1 subunit alpha	7.7084	
	Farnesyltransferase, CAAX box, alpha	7.4005	

Supplementary Figure S3:

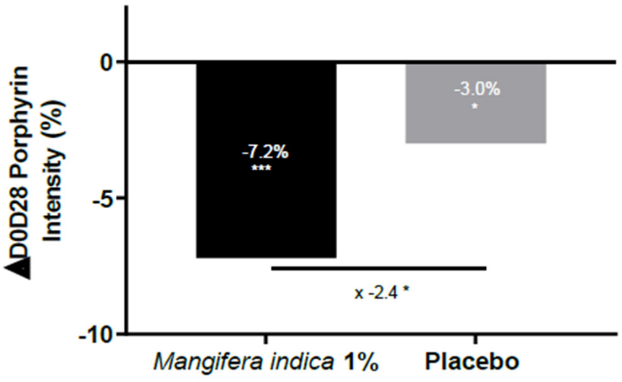

Figure S3: Porphyrin intensity analysis by VISIA® CR 2.3 on Caucasian volunteers (cheek and nose areas) after 28 days of application of cream containing *M. indica* at 1% or placebo.
