# Supplementary material for: Action of Mangifera indica Leaf Extract on Acne-Prone Skin through Sebum Harmonization and Targeting C. acnes
Source: Molecules. 2022 Jul 26;27(15):4769. doi: 10.3390/molecules27154769 (PMC9331558; doi:10.3390/molecules27154769)
Supplement: Supplementary file 1 [file molecules-27-04769-s001.zip › Supplementary material_19072022.pdf]

Action of *Mangifera indica* leaf extract on the *C. acnes* lipase activity: a biological evaluation

SUPPLEMENTARY MATERIALS

Characterization and quantification of phytomarkers in *Mangifera indica* extract by HPLC-UV

Characterization and quantification is performed against mangiferin, iriflophenone-3-C-beta-glucoside and penta-O-galloyl-beta-D-glucose standards after separation on C18 reverse phase column with UV detection at 315 nm for mangiferine and 295 nm for both benzophenones and gallotanin. Maclurin-3-C-beta-glucoside is quantified with the response factor of iriflophenone-3-C-beta-glucoside. The apparatus is a HPLC Agilent 1260, equipped with high sensitive UV-vis detector (DAD, optical path length 10 cm), column (Atlantis T3, 5µm 4.6x150mm), temperature (30°C), Flow rate: 1.0mL/min, injection volume: 2µL, detection with high sensitive cell (DAD) at 315nm for mangiferin and 295nm for Maclurin and Iriflophenone, gradient: (B) Acetonitrile and (A) water with 1% of acetic acid according the table below:

Time (min)	A (%)	B (%)
0	95	5
15	75	25
25	50	50
26	0	100
30	0	100

For sample preparation, accurately weigh around 500 mg of the propanediol mangifera extract into 50 ml volumetric flask. Dilute with diluent and sonicate 2 minutes before filtration through 0.45 PTFE filter. For standard preparation for calibration curves conditions: mangiferin is purchased from extrasynthese, ref 0431S, purity 98.2%. preparation of a solution of 5mg in 25mL of solvent EtOH/H2O/DMSO (80/10/10, v/v/v). preparate dilutions into EtOH in order to get 6 points of calibration in the range of 5-200 µg/mL.Iriflophenone-3-C-beta-glucoside is purchased from Sigma-aldrich, ref 91267, purity 97.5%. preparation of a solution of 2mg in 50mL of solvent EtOH/H2O/DMSO (80/10/10, v/v/v). preparate dilutions into EtOH in order to get 6 points of calibration in the range of 5-50 µg/mL. Pentagalloyl-O-beta-D-glucose is purchased from Greenpharma, ref amb6296489. preparation of a solution of 2mg in 10mL of solvent EtOH/H2O/DMSO (80/10/10, v/v/v). preparate dilutions into EtOH in order to get 6 points of

calibration in the range of 8-200 µg/mL. Calibration graphs were obtained by plotting the peak area vs the concentration of the standard solutions.

In vitro **lipogenesis inhibition assay**

Analysis of the lipid content (Bodipy® labeling)

At the end of the incubation, the cells were rinsed, fixed and permeabilized. The lipid droplets contained in the cells were then labeled using a specific Bodipy® fluorescent lipid probe which mainly detects neutral lipids. In parallel, the cell nuclei were stained using a Hoechst 33258 (bis-benzimide) solution. The acquisition of the images was performed using INCell Analyzer™ 1000 (GE Healthcare). Ten photos were taken per well for each labeling (×20 objective lens). The labeling was quantified by measuring the fluorescence intensity normalized to the total number of cells (integration of numerical data with the Developer Toolbox 1.5, GE Healthcare software). The fluorescence intensity was analyzed exclusively in the lipid droplets (image analysis program based on object segmentation). Therefore, the image analysis did not consider a non-specific fluorescent background signal, which can be frequently observed in high-confluence cultures of SEBO662AR.

Evaluation of the anti-lipase activity of *Mangifera indica* on severe *C. acnes* phylotype

Culture of Severe acne C. acnes phylotype (IA1, ARCC 6919), treatment and oleic acid quantification

Severe *C. acnes* phylotype (IA1, ARCC 6919) was cultured in reinforced clostridial broth as recommended by ATCC at 25°C. PH was adjusted at 6.8 ± 0.2. The bacteria were incubated for 2 days in order to initiate the growth. During the exponential phase, the triolein was added at 1% to the culture with *Mangifera indica* at 2% (or not, control condition). At the end of the incubation, the colonies number was counted and quantified.

The medium of each condition was collected in order to identify and quantify the lipids present at the end of culture. Briefly, the tubes containing the samples were thawed at room temperature then centrifuged for 5 min at 20,000 g at 4°C. The liquid was then collected and placed into a new tube. A mixture of 1 ml of purified water and 3 ml of chloroform/methanol was added. The mixture was stirred at room temperature for 1 hour then centrifuged for 5 min at 3500 rpm. The aqueous superior phase was collected and placed into a new tube. 2 ml of chloroform was added, and the mixture was stirred for 10 min at room temperature, then centrifuged again for 5 min at 3500 rpm.

The organic inferior phase was collected and pooled with the previous organic phase. The organic phases were then evaporated until dryness under nitrogen at 50°C and dissolved into chloroform/methanol mixture. The samples were finally diluted for analysis. The oleic acid quantification was realized with a GC system (7890A from Agilent) coupled with an MS system (5975C Inert XL EI/CI MSD from Agilent).

***Ex vivo* lipogenesis characterization**

Lipids extraction

The tissues were extracted with an aqueous cocktail overnight at 4°C. They were then treated to take off the epidermis. A specific technic was used to recover the glands attached to the hair shafts (proprietary of the CRO).
After rinsing in an isotonic medium, the epidermis and its appendages were transferred to a watch glass. The sebaceous glands attached to the hair shaft of hair follicles were cut at the level of the pilosebaceous canal and deposited against the walls of the recovery vials. The sebaceous glands were recovered after dilaceration of the epidermal fragments. The glands were collected in a batch of 10 units. The glands were then washed to eliminate any contaminations of non-sebaceous origin. Glands were pooled and extracted using a Bligh Dyer protocol using water, methanol and chloroform. The lipidic residue was then assayed using 2 methods.

Lipids analysis and quantification

The first one focus on Free Fatty acids and the second for Squalene, Cholesterol and Glycerides.

For FFA, residue was solubilized with chloroform and methanol. After a centrifugation, the supernatant was methylated by incubation with 1 N H_2SO_4 in methanol. The fatty acid methyl esters were then extracted in n-hexane and analyzed on an Agilent 7890A temperature controlled gas chromatograph (Santa Clara, CA, USA) equipped with a 30 m SLB-IL111 fused silica capillary with a film thickness of 0.2 mm, coupled to an Agilent 5975C mass spectrometer.

The injection port temperature was 250°C and the detector port temperature was 255°C. The carrier gas was helium.

For neutral lipids, residue was solubilized with à mixture of chloroform and methanol. After a centrifugation, the supernatant was evaporated and dissolved in dichloromethan to be analyzed on an Agilent 7890A temperature controlled gas chromatograph (Santa Clara, CA, USA) equipped with

a 30 m Zebron 5HT fused silica capillary with a film thickness of 0.25 mm, coupled to an Agilent 5975C mass spectrometer (Inert XL EI/CI MSD). The injection port temperature was 315°C and the detector port temperature was 280°C. The carrier gas was helium.

Clinical investigations - Metagenomic analysis

16S rRNA gene sequencing and data analysis

Sequencing was performed with the MiSeq device (Illumina, Inc., San Diego, CA, USA) through a 500 cycles paired-end run, targeting the V3V4 16S variable regions using the following primers: 16S-Mi341F forward primer 5'-CCTACGGGNGGCWGCAG-3' and 16S-Mi805R reverse primer 5'-GACTACHVGGGTATCTAATCC-3', producing about 460 bp amplicons.

PCR1s were performed as follows: 8 µl of template DNA (0.2 ng) were mixed with 5 µl of each reverse and forward primers (1 µM), 5 µl of KAPA HiFi Fidelity Buffer (5×), 0.8 µl of KAPA dNTP Mix (10 mM each), 0.7 µl of RT-PCR grade water (Ambion), and 0.6 µl of KAPA HiFi hotstart Taq (1 U/µl), for a total volume of 25 µl. Each amplification was duplicated, and duplicates were pooled after amplification. PCR1 cycles consisted of 95°C for 3 min and then 32 cycles of 95°C for 30 s, 59°C for 30 s, and 72°C for 30 s, followed by a final extension at 72°C for 3 min, with a BioRad CFX1000 thermocycler. Negative and positive controls were included in all steps to check for contamination. All duplicate pools were controlled by gel electrophoresis, and amplicons were quantified using fluorometry.

Libraries ready for analysis were then produced following the Illumina guidelines for 16S metagenomics libraries preparation. Briefly, the PCR1 amplicons were purified and controlled using an Agilent 2100 Bioanalyzer (Agilent Technologies, CA, USA). To enable the simultaneous analysis of multiple samples (multiplexing), Nextera® XT indexes (Illumina) were added during PCR2 using 15–30 ng of PCR1 amplicons. PCR2 cycles consisted of 94°C for 1 min and then 12 cycles of 94°C for 60 s, 65°C for 60 s, and 72°C for 60 s, followed by a final extension at 72°C for 10 min. Indexed libraries were purified, quantified and controlled using an Agilent 2100 Bioanalyzer. Validated indexed libraries were pooled in order to obtain an equimolar mixture.

The run (500 cycles) was achieved on MiSeq sequencer (Illumina) using the MiSeq Reagent Kit v3 600 cycles (Illumina). The sequencing run produced an output of 12.7 million paired-end reads of 250 bases, i.e., up to 3.2 Gigabases. Givaudan performed the libraries and the MiSeq run at the GeT-PlaGe platform (INRA, Auzeville, France).

After the MiSeq run, raw data sequences were demultiplexed and quality-checked to remove all the reads with ambiguous bases. Indexes and primers sequences were removed with cutadapt (v1.9; http://cutadapt.readthedocs.io/en/stable/index.html), and reads with fastq score lower than 28 were trimmed. The forward and reverse sequences were paired using bbmerge (https://jgi.doe.gov/data-and-tools/bbtools/). Samples with <5000 paired sequences were discarded. The remaining paired sequences were then treated using an in-house pipeline that uses vsearch [1] to remove chimeras and amplicons with PCR errors. Sequences were then split into Operational Taxonomic Units (OTUs, a cluster of similar sequence variants of the 16S rRNA marker gene sequence) at a 1% dissimilarity level using swarm (v2.6).[2] Unique amplicons were mapped to the SILVA SSU Ref NR 99 (non-redundant) database (release 132; https://www.arb-silva.de/) for taxonomic assignation using the RDP classifier. [3] Data normalization and analyses were done using R statistical computing environment (v3.2.0; https://www.r-project.org - R core team (2014) using Bioconductor package (mainly Phyloseq, DESeq2 and Vegan libraries; http://www.bioconductor.org).

Data were then compared using Wilcoxon's test for paired samples. Due to multiple testing, the p-value was adjusted using false discovery rate (FDR) correction.[4]

REFERENCES

1. Rognes T, Flouri T, Nichols B, Quince C, Mahé F. VSEARCH: a versatile open source tool for metagenomics. PeerJ. 2016 Oct 18;4:e2584. doi: 10.7717/peerj.2584.

2. Mahé F, Rognes T, Quince C, de Vargas C, Dunthorn M. Swarm v2: highly-scalable and high-resolution amplicon clustering. PeerJ. 2015;3:e1420. Published 2015 Dec 10. doi:10.7717/peerj.1420

3. Wang Q, Garrity GM, Tiedje JM, Cole JR. Naive Bayesian classifier for rapid assignment of rRNA sequences into the new bacterial taxonomy. Appl Environ Microbiol. 2007;73(16):5261-5267. doi:10.1128/AEM.00062-07

4. Benjamini, Yoav, and Yosef Hochberg. "Controlling the False Discovery Rate: A Practical and Powerful Approach to Multiple Testing." Journal of the Royal Statistical Society. Series B

(Methodological), vol. 57, no. 1, [Royal Statistical Society, Wiley], 1995, pp. 289–300, http://www.jstor.org/stable/2346101.
